# Venomous Secretions from Marine Snails of the Terebridae Family Target Acetylcholine Receptors

**DOI:** 10.3390/toxins5051043

**Published:** 2013-05-21

**Authors:** Yvonne Kendel, Christian Melaun, Alexander Kurz, Annette Nicke, Steve Peigneur, Jan Tytgat, Cora Wunder, Dietrich Mebs, Silke Kauferstein

**Affiliations:** 1 Institute of Legal Medicine, University of Frankfurt, Kennedyallee 104, Frankfurt D-60596, Germany; E-Mails: yvonne.kendel@web.de (Y.K.); alexander.kurz.research@online.ms (A.K.); wunder@med.uni-frankfurt.de (C.W.); 2 Biodiversity and Climate Research Center (BiK-F), Senckenberganlage 25, Frankfurt D-60325, Germany; E-Mails: christian.melaun@senckenberg.de (C.M.); mebs@em.uni-frankfurt.de (D.M.); 3 Senckenberg Gesellschaft für Naturforschung, Senckenberganlage 25, Frankfurt D-60325, Germany; 4 Max-Planck-Institute of Experimental Medicine, Hermann-Rein-Str. 3, Göttingen D-37075, Germany; E-Mail: anicke@gwdg.de; 5 Laboratory of Toxicology and Pharmacology, University of Leuven, Campus Gatshuisberg, Herestraat 49, Leuven B-3000, Belgium; E-Mails: steve.peigneur@pharm.kuleuven.be (S.P.); jan.tytgat@pharm.kuleuven.be (J.T.)

**Keywords:** Terebridae venom, gland extracts, acetylcholine receptors, potassium channels, sodium channels

## Abstract

Venoms from cone snails (Conidae) have been extensively studied during the last decades, but those from other members of the suborder Toxoglossa, such as of Terebridae and Turridae superfamilies attracted less interest so far. Here, we report the effects of venom and gland extracts from three species of the superfamily Terebridae. By 2-electrode voltage-clamp technique the gland extracts were tested on *Xenopus* oocytes expressing nicotinic acetylcholine receptors (nAChRs) of rat neuronal (α_3_β_2_, α_3_β_4_, α_4_β_2_, α_4_β_4_, α_7_) and muscle subtypes (α_1_β_1_γδ), and expressing potassium (Kv1.2 and Kv1.3) and sodium channels (Nav1.2, 1.3, 1.4, 1.6). The extracts were shown to exhibit remarkably high inhibitory activities on almost all nAChRs tested, in particular on the α_7_ subtype suggesting the presence of peptides of the A-superfamily from the venom of *Conus* species. In contrast, no effects on the potassium and sodium channels tested were observed. The venoms of terebrid snails may offer an additional source of novel biologically active peptides.

## 1. Introduction

Marine gastropods of the suborder Toxoglossa comprise three major superfamilies: the cone snails (Conidae, about 800 species), the auger snails (Terebridae, 300 to 400 species) and the turrids (Turridae, more than 10,000 species) [1,2,3]. These are predatory, carnivorous snails which capture their prey, *i.e.*, worms and to less extent snails and fish, by injecting venom. Over the last decades, biochemical and pharmacological research focussed mainly on cone snails (*Conus* spp.) leading to the discovery of a great variety of biologically active peptides and proteins that affect neurotransmissions by acting on nervous structures such as ligand- and voltage-gated ion channels, transporters and receptors [4,5,6]. However, in contrast to the impressive progress made in understanding the toxinology and ecology of cone snails, very few studies have been performed on the venoms of Terebridae and Turridae. 

Several peptides have been identified in the venom of two terebrids, *Terebra subulata* [7] and *Hastula hectica* [8], and in the venom of some turrid snails, *Polystira albida* [9,10], *Lophiotoma olangoensis* [11], *Gemmula speciosa* [12], *G. periscida* and *Clathurella cincta* [13]. These disulfide-rich peptides consist of 11 to 41 amino acids and show features similar to those of conopeptides. The cysteine framework of peptides from the venom of *Terebra subulata* and *Hastula hectica* was found to be similar to that of peptides of the O-superfamily of *Conus* species [7,8]. However, the signal sequence of their precursor region shows no homology to that of O-superfamily conotoxins. Peptides, *i.e.*, teretoxins from *Hastula hectica*, exhibit divergent signal sequences, although the cysteine frameworks of the mature peptides are consistent with those for O and P conotoxins. Moreover, terebrid peptides seem to be less post-translationally modified than conotoxins [7]. However, the biological activity of terebrid peptides is still not known. Some of these venom components have been tested on the nematode *Caenorhabditis elegans*, which showed an uncoordinated twisting syndrome after injection, but no unusual behavioural symptomatology was observed in mice following intracranial application [7,8].

In the present study, the effects of gland extracts obtained from three Terebridae species were tested on nicotinic acetylcholine receptors (nAChR) as well as on voltage-gated Na^+^- and K^+^-channels expressed in *Xenopus* oocytes using voltage-clamp technique. The results indicate the presence of peptides in the gland secretions, most likely the venom, which are predominantly selective for nAChRs.

## 2. Results

### 2.1. Venom Apparatus of the Terebridae

**Figure 1:**
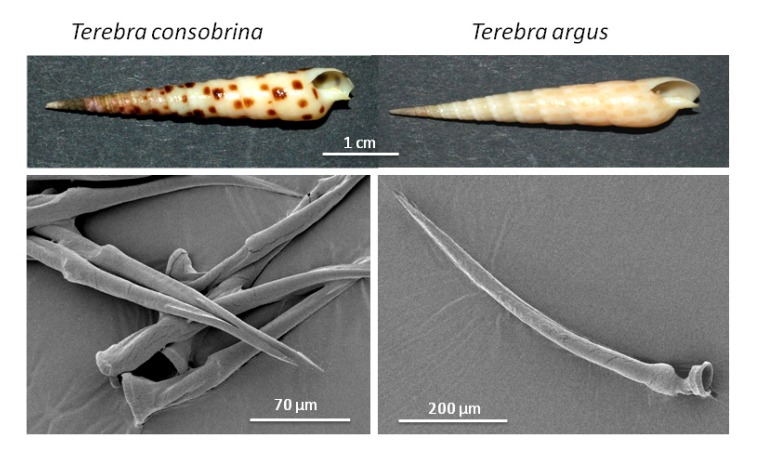
SEM-pictures of radula teeth from two Terebridae species, *Terebra consobrina* and *T. argus*.

The two *Terebra* species examined in this study, namely, *Terebra argus* and *T. consobrina*, have a venom apparatus which is similar to that of *Conus* snails consisting of a radular sac, a venom duct and venom bulb. Characteristic harpoon-like, hypodermic radula teeth were detected in both species by dissection of the radula sac (Figure 1). 

No such radula teeth were found in *Oxymerismaculata* (formerly *Acus*). However, the tissue excised from the foregut used for extraction was found to contain a gland, most probably a salivary gland. 

### 2.2. Electrophysiological Analysis of the Venom Gland Extracts

**Figure 2:**
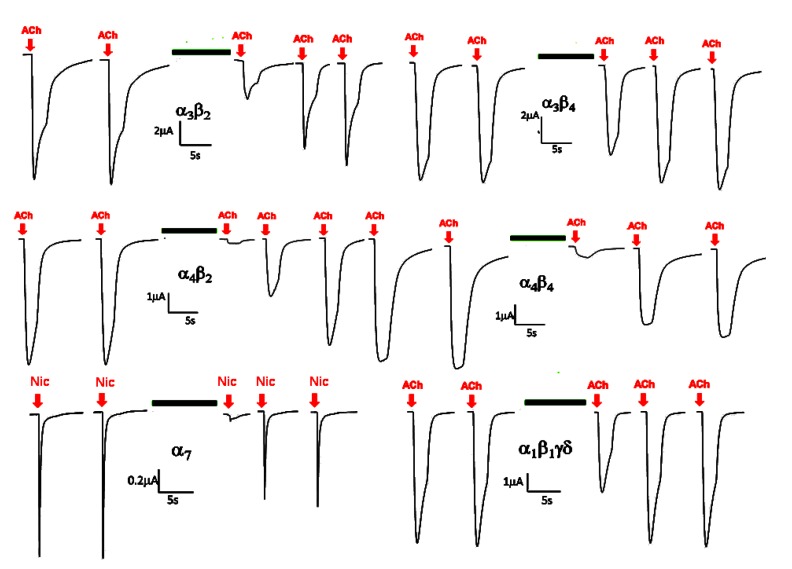
Effects of *Terebra consobrina* venom gland extract (12.9 μg) on six nicotinic acetylcholine receptor (nAChR) subtypes. 100 µM ACh or nicotine (in the case of the α_7_) were applied for 2 s in 4 min intervals. Two current responses before application of gland extracts, one response directly after extract application (horizontal bar, 3 min incubation) and two subsequent responses after washout of the extracts are shown for each subtype.

Gland extracts were prepared by homogenizing the glandular tissue from the three Terebridae species in 10% acetic acid followed by centrifugation and lyophilization of the supernatant and were qualitatively tested on nAChRs. Remarkable blocking activity between 50% and 100% on neuronal as well as on muscle nAChR subtypes was detected in the gland extracts from the three species (Table 1, Figure 2). In the species *Oxymeris* and *Terebra consobrina* even 10-fold dilutions of the stock solution caused significant inhibitory effects on the α_4_β_2_ and α_7_ receptors. The muscle nAChR subtype, α_1_β_1_γδ, was mainly affected by the *Oxymeris* gland extract, but less by the extract from *T. consobrina*. In contrast to these specific blockades, none of the gland extracts showed effects on common potassium (Kv1.2 and Kv1.3) or sodium channels (Nav1.2, 1.3, 1.4, 1.6).

**Table 1:** Blocking activity of gland extracts from Terebridae species, undiluted (10 to 13 µg) and diluted 1:10, on neuronal (α_3_β_2_, α_3_β_4_, α_4_β_4_ and α_7_) and muscle subtype nAChRs (α_1_β_1_γδ) expressed in *Xenopus* oocytes using voltage-clamp technique.

	*Oxymeris maculata*	*Terebra argus*	*Terebra consobrina*
α_3_β_2_	++	+/−	++
1:10	+	−	+
α_3_β_4_	+	−	+
1:10	−	−	−
α_4_β_2_	+++	+	+++
1:10	+	−	+
α_4_β_4_	+++	+/−	++
1:10	−	−	−
α_7_	+++	+	+++
1:10	+	−	+
α_1_β_1_γδ	+++	−	+
1:10	+	−	−

+++ indicates complete blockage; ++ more than 80%; **+** 50% to 80%; +/− less than 50%; − no blockage.

In a preliminary mass-spectrometric analysis (LC-MS-TOF), compounds representing molecular weights between 1138 and 4446 Da were identified in the extracts, predominating in the range of 1.1 and 2.1 kDa. Lack of material prevented further studies such as HPLC-fractionation.

## 3. Discussion

The present pharmacological study on extracts of venom glands (*Terebra argus*, *T. consobrina*) and of other, probably salivary glandular tissue (*Oxymeris maculata*) from three Terebridae species revealed high inhibitory activities to nAChRs predominantly of the neuronal subtype, whereas no effects on K^+^- and Na^+^-channels were found. The observed effects are similar to those produced by α-conopeptides from the venom of cone snails which consist of 13 to 19 amino acids cross-linked by two disulfide bonds and exhibit molecular weights between 1.4 and 2.1 kDa [14]. For example, in the terebrid gland extracts some components were found to be in the same order of magnitude (1449 Da in *Terebra consobrina*, 1443 Da in *Oxymeris maculata* gland extracts, respectively) like that of the GI α-conopeptide (1437 Da) from the venom of *Conus geographus* [15].

The inhibitory activity of the gland extracts on the neuronal nAChRs follows the order: α_7_ > α_4_β_2_ > α_4_β_4_ > α_3_β_2_ > α_3_β_4_. The affinity of peptides in the extracts to nAChRs containing α_4_ subunits seems to be higher than to receptors containing α_3_ subunits, when combined with the β_2_ subunit. In the α_3_β_4_ subtype inhibition is low. α_4_-containing nAChR-subtypes are not predominantly targeted by most cone snail venoms and no conopeptide exhibiting high affinity to these receptors has been identified, so far [16,17]. This might suggest the presence of pharmacologically active peptides with a novel selectivity profile in the terebrid gland extracts. It is interesting to note that only the gland extract from *Oxymeris maculata* produced a complete blockage of the muscle-type receptor α_1_β_1_γδ. However, more conclusive results may be obtained when peptides from the extracts are isolated and tested on these receptors. 

The absence of effects on K^+^- and Na^+^-channels could be due to: (i) the complete absence of peptides modulating K^+^- and Na^+^-channels; (ii) the presence of peptides with only low affinity to these ion channels; and/or (iii) the low concentration in the extracts of peptides of interest. 

Recently, Castelin *et al*. [18] demonstrated that there exists a great disparity of the terebrid foregut anatomy such as the presence or absence of the proboscis and venom glands as well as a great variety of radula structures. In the species of the present study, venom glands and harpoon-like radula teeth were present in the two *Terebra* species, but absent in *Oxymeris maculata*, which, on the other hand, has salivary glands in the foregut suggesting that the active components in the extract originate from this gland. Although salivary glands were considered to play a minor role only in prey envenoming, Biggs *et al*. [19] have found that α-conopeptides are also expressed in the salivary gland of *Conus pulicarius*. Based on the analysis of the terebrid molecular phylogeny, five [20,21], and in a recent study [18] six terebrid lineages have been distinguished. Among these, *Oxymeris* (*Acus*) species were supposed to have independently lost its venom apparatus. 

Major targets of these gland secretions seem to be nAChRs. This is in good agreement with the observation that the inhibition of nAChRs by peptides acting like those of the A-superfamily of *Conus* species, is a very effective mode to rapidly paralyze prey and that the nAChR is a target of a wide variety of venomous animals and poisonous plants. Whether further glandular components of the Terebridae are present acting for example on neuronal calcium channels requires further investigation.

## 4. Experimental Section

### 4.1. Materials

Terebridae specimens (*Oxymeris maculata*, *Terebra argus*, *T. consobrina*) were collected in the reefs of Cebu, Philippines. All specimens were kept frozen at −20 °C until preparation. The venom ducts (*Terebra* species) or glandular tissue from the foregut (*Oxymeris*) were dissected and placed in 10% acetic acid. Extracts were prepared by homogenizing the glands in 10% acetic acid, separating the mixture by centrifugation at 3000 rpm for 15 min and recovering the supernatant that was lyophilized and stored at −20 °C.

### 4.2. Electrophysiology

nAChR cDNAs were provided by J. Patrick (Baylor College of Medicine, Houston, TX, USA) and subcloned into the oocyte expression vector pNKS2. cRNA was synthesized with the SP6 mMessage mMachine kit (Ambion, Austin, TX, USA) and *Xenopus laevis* (Nasco International, Fort Atkinson, WI, USA) oocytes were injected with 50 nL aliquots of cRNA (0.5 mg/mL). One to three days after injection two-electrode voltage clamp recordings were performed on *Xenopus* oocytes. The activity of the extracts dissolved in 1.0 mL ND96 solution (96 mM NaCl, 2 mM KCl, 1 mM CaCl_2_, 1 mM MgCl_2_, and 5 mM Hepes, pH 7.4) was investigated qualitatively on nicotinic acetylcholine receptors (nAChRs) of rat neuronal (α_3_β_2_, α_3_β_4_, α_4_β_2_, α_4_β_4_, α_7_) and muscle subtypes (α_1_β_1_γδ). Current responses to 100 µM acetylcholine or 100 µM nicotine (used for the α_7_ subtype) were measured at a holding potential of −70 mV using a Turbo Tec 05X Amplifier (NPI Electronic, Tamm, Germany) and Cell Works software. Currents were filtered at 200 Hz and digitized at 400 Hz. The perfusion medium was automatically switched between ND96 with or without agonist using a custom-made magnetic valve system. A fast and reproducible solution exchange (<300 ms) was achieved using a 50 µL funnel-shaped oocyte chamber combined with a fast solution flow fed through a custom-made manifold mounted immediately above the oocyte. Agonist pulses were applied for 2 s at 4 min intervals. After each application, the cell was superfused for 1 min with agonist-free ND96, before the flow was stopped for 3 min and the gland extract was immediately mixed into the bath. Each extract dilution was tested on at least three oocytes. 

For testing effects of the gland extracts on Na^+^- and K^+^-channels, two-electrode voltage-clamp recordings were performed at room temperature (18–22 °C) using a Geneclamp 500 amplifier controlled by a pClamp data acquisition system (Molecular Devices, Sunnyvale, CA, USA). Whole cell currents from *Xenopus* oocytes were recorded expressing Na^+^- and K^+^-channels 1–4 days after injection. Bath solution composition was ND96 and HEPES, 5 mM (pH 7.4) or HK (96 mM NaCl, 2 mM KCl, 1.8 mM CaCl_2,_ 2 mM MgCl_2_) and HEPES, 5 mM (pH 7.4). Voltage and current electrodes were filled with 3 M KCl solution. Resistances of both electrodes were kept between 0.5 and 1.5 MΩ. The elicited currents were filtered at 1 kHz and sampled at 0.5 kHz (for potassium currents) or at 20 kHz (for sodium currents) using a four-pole low-pass Bessel filter. Leak subtraction was performed using a −P/4 protocol. K_V_1.2 and K_V_1.3 potassium currents were evoked by 500 ms depolarizations to 0 mV followed by a 500 ms pulse to −50 mV. From a holding potential of −90 mV, sodium current traces were evoked by 100 ms depolariarions to *V*_max_ (the voltage corresponding to maximal sodium current in control conditions). In order to investigate the current-voltage relationship, current traces were evoked by 10 mV depolarization steps from a holding potential of −90 mV.

## 5. Conclusions

In contrast to the extensively studied venom from cone snails, the pharmacological activities of Terebridae venoms are completely unknown. This first study on three Terebridae species demonstrates that venom or salivary gland extracts are producing distinct inhibitory effects on a variety of neuronal and muscle nAChR subtypes. Venoms from this snail superfamily represent an untapped resource and offer the opportunity to discover novel pharmacologically active peptides with pharmaceutical and therapeutic potentials. 
